# Development and Validation of a Nomogram Based on Noninvasive Liver Reserve and Fibrosis (PALBI and FIB-4) Model to Predict Posthepatectomy Liver Failure Grade B-C in Patients with Hepatocellular Carcinoma

**DOI:** 10.1155/2021/6665267

**Published:** 2021-04-07

**Authors:** Wenhui Zhong, Feng Zhang, Kaijun Huang, Yiping Zou, Yubin Liu

**Affiliations:** ^1^Department of Hepatobiliary Surgery, Guangdong Provincial People's Hospital, Guangdong Academy of Medical Sciences, Guangzhou 510000, Guangdong, China; ^2^Shantou University of Medical College, Shantou 515041, China

## Abstract

Hepatectomy is currently one of the most effective treatments for hepatocellular carcinoma (HCC). However, postoperative liver failure (PHLF) is a serious complication and the leading cause of mortality in patients with HCC after hepatectomy. This study attempted to develop a novel nomogram based on noninvasive liver reserve and fibrosis models, platelet-albumin-bilirubin grade (PALBI) and fibrosis-4 index (FIB-4), able to predict PHLF grade B-C. This was a single-centre retrospective study of 574 patients with HCC undergoing hepatectomy between 2014 and 2018. The independent risk factors of PHLF were screened using univariate and multivariate logistic regression analyses. Multivariate logistic regression was performed using the training set, and the nomogram was developed and visualised. The utility of the model was evaluated in a validation set using the receiver operating characteristic (ROC) curve. A total of 574 HCC patients were included (383 in the training set and 191 for the validation set) and included PHLF grade B-C complications of 14.8, 15.4, and 13.6%, respectively. Overall, cirrhosis (*P* < 0.026, OR = 2.296, 95% confidence interval (CI) 1.1.02–4.786), major hepatectomy (*P*=0.031, OR = 2.211, 95% CI 1.077–4.542), ascites (*P*=0.014, OR = 3.588, 95% 1.299–9.913), intraoperative blood loss (*P* < 0.001, OR = 4.683, 95% CI 2.281–9.616), PALBI score >−2.53 (, OR = 3.609, 95% CI 1.486–8.764), and FIB-4 score ≥1.45 (*P* < 0.001, OR = 5.267, 95% CI 2.077–13.351) were identified as independent risk factors associated with PHLF grade B-C in the training set. The areas under the ROC curves for the nomogram model in predicting PHLF grade B-C were significant for both the training and validation sets (0.832 vs 0.803). The proposed nomogram predicted PHLF grade B-C among patients with HCC with a better prognostic accuracy than other currently available fibrosis and noninvasive liver reserve models.

## 1. Introduction

Hepatocellular carcinoma (HCC) is the sixth highest common malignancy and ranks fourth among cancer-related deaths globally [1]. Hepatectomy is the preferred treatment for liver cancer patients who are diagnosed with resectable and early-stage HCC [2, 3]. Although safety and short-term outcomes after surgery have been enhanced as a result of advanced surgical techniques and preoperative management, posthepatectomy liver failure (PHLF) remains the most worrisome complication with high morbidity (0.7–34%) and mortality (50%), which will ultimately prolong hospitalisation, increase hospital costs, and impair quality of life in patients undergoing hepatectomy [4–7].

Several predicting models of PHLF such as the Child–Pugh grade, the model for end-stage liver disease (MELD) score, albumin-bilirubin (ALBI) score, platelet-albumin-bilirubin (PALBI) score, aspartate aminotransferase to platelet ratio index (APRI), and fibrosis-4 index (FIB-4) have been reported [8–12], but the performances of the above models still have drawbacks. The Child–Pugh grade is still widely used to provide an assessment of liver function in clinical work. However, the essential component in this model such as ascites or hepatic encephalopathy lacks objective standards, and the limitations have been suggested in previous studies [13]. MELD is a scoring tool widely used to evaluate the severity of end-stage liver disease and efficacy of transplantation [14] and was original developed to predict survival in patients undergoing a transjugular intrahepatic portosystemic shunt (TIPS) [15]. However, the accuracy of prediction decreases for postoperative morbidity and mortality for patients undergoing hepatectomy, cirrhosis with ascites, or hepatic encephalopathy [16, 17]. Furthermore, MELD may not be applicable in patients with PHLF [10, 18]. The ALBI grading system was created in 2015 as a convenient and simple tool for assessing liver function in patients with HCC [13], which not only eliminated some subjective variables but also reduced errors due to numerous indexes. Portal hypertension (PH) imposes a high risk of postoperative complication and is closely related to mortality [19]. However, the ALBI grading system does not have any factors associated with PH. The blood platelet count (PLT) was used as an improved marker for PH and added to the PALBI [20], which has shown wonderful power in assessing preoperative liver reserve function and predicting survival [21]. Despite these advantages, the PALBI score still needs to evaluate the accuracy in predicting PHLF by more independent research and with larger amounts of data. The level of hepatic fibrosis is associated with preoperative liver dysfunction and has been discovered to be one of the most important risk factors for the development of liver failure and HCC causing death [22]. The APRI and FIB-4 are noninvasive and reliable fibrosis scores based on laboratory parameters with an impressive performance in assessing significant fibrosis or cirrhosis [12]. However, the APRI score only includes two quantitative variables, serum aspartate transaminase (AST) and PLT counts, which have no ceiling effect and are not optimal factors of liver function. Similarly, the FIB-4 index has obvious limitations on the ability to predict liver fibrosis due to underlying disease, and its predicted performance for late-stage fibrosis is inadequate. Therefore, the above conventional models have only focused on preoperative liver reserve function or fibrosis severity without comprehensively considering PHLF-related risk factors, and the accuracy of predicting PHLF is still controversial.

In this study, we retrospectively used a large patient dataset to construct and validate a novel nomogram model combining the PALBI and FIB-4 scores to predict PHLF grade B-C. We then assessed the accuracy of nomogram and conventional model among PHLF patients subjected to hepatectomy.

## 2. Materials and Methods

### 2.1. Study Patients

This study retrospectively collected data from 574 consecutive patients who underwent hepatectomy for pathologically proven HCC at the Guangdong Provincial People's Hospital from January 2014 to December 2018. Patients were randomly divided into the training set (*n* = 383) and validation set (*n* = 191). This retrospective study adhered to the provisions of the Declaration of Helsinki. The need for patient written informed consent was waived given the study's retrospective nature. The Research Ethics Committee of our hospital approved the retrospective study.

Patients qualified for inclusion met the following criteria: (1) age 18 to 85 years, (2) preoperative Child–Pugh grade A or B, (3) subjected to R0 resection, and (4) pathologically confirmed as HCC. Patients were excluded for the following reasons: (1) a history of preoperative anticancer therapies such as chemotherapy, radiotherapy, or transcatheter arterial chemoembolisation (TACE); (2) missing important clinical information; and (3) presence of other cancers except for HCC.

### 2.2. Definitions

PHLF was defined based on the International Study Group of Liver Surgery (ISGLS) definition [23]: serum international normalised ratio (INR) and serum bilirubin concentration increased on or after postoperative day 5. PHLF grade A required no change in the clinical treatment of patients, grade B causing a deviation from the regular clinical management but required noninvasive treatment, and grade C required invasive treatment. In this study, grade B-C was defined as severe PHLF. Clinically significant portal hypertension (CSPH) was diagnosed as the presence of oesophageal/gastric varices by endoscopy and/or a platelet count below 100 × 10^9^/L associated with splenomegaly [24]. Cirrhosis was diagnosed based on histopathological findings. Major hepatectomy was defined as 3 or more liver segmental resections [25]. Ascites was diagnosed by imaging tests such as computed tomography or abdominal ultrasound examination [26].

### 2.3. Calculation of Child–Pugh, ALBI, MELD, APRI, PALBI, and FIB-4 Scores

The Child–Pugh score was calculated by adding a point for three continuous and two categorical factors: serum albumin, total bilirubin, prothrombin time, hepatic encephalopathy, and ascites. Child–Pugh grading was A (5-6 point), B (7–9 point), or C (10–15 point) [27]. The ALBI score formula was as follows: ALBI score = (log^10^ bilirubin (*μ*mol/L) × 0.66) + (albumin (g/L) × (−0.085)). The ALBI grades were divided into grade 1 (score ≤−2.60), grade 2 (score >−2.60 to ≤−1.39), and grade 3 (score >−1.39) [13]. The MELD score was calculated as 9 : 57 × ln (creatinine) + 3.78 × ln (total bilirubin) + 11.2 × ln (INR) + 6.4 [8]. The APRI score was calculated as ((AST (U/L)/ULN)/PLT count (10^9^/L)) × 100 [11]. The PALBI grades were calculated using the following algorithm: PALBI score = 2.02 × log^10^ bilirubin − 0.37 × (log^10^ bilirubin) − 0.04 × albumin − 3.48 × log^10^ PLT + 1.01 × (log^10^ PLT). The PALBI grades were divided into grade 1 (score ≤−2.53), grade 2 (score >−2.53 to ≤−2.09), and grade 3 (score >−2.09) [21]. We intentionally categorised PALBI grade 1 into the low PALBI group and grade 2-3 into the high PALBI group. The FIB-4 score was calculated using the following algorithm: FIB-4 score = AST (U/L) × age (years)/(platelet count (×10^9^/L) × ALT (U/L)^1/2^). The FIB-4 grades were divided into grade 1 (score <−1.45), grade 2 (score ≥−1.45 to ≤3.25), and grade 3 (score >−3.25). We intentionally categorised FIB-4 grade 1 into the low FIB-4 group and grades 2-3 into the high FIB-4 group [28].

### 2.4. Clinical Examination Collection and Hepatectomy

All the preoperative variables based on the results of the latest blood sampling period to surgery including the INR, creatinine, serum albumin, alanine aminotransferase (ALT), AST, alkaline phosphatase (ALP), *γ*-glutamyl transferase (*γ*-GGT), alpha fetoprotein (AFP), and surgical variables (intraoperative blood loss and extent of hepatectomy), tumour characteristics (largest tumour size, number of tumour, and microvascular invasion), cirrhosis, PH, 4 noninvasive liver reserve models (Child–Pugh, ALBI, MELD, APRI, and PALBI), and 1 fibrosis model (FIB-4) were routinely collected. All hepatectomy procedures were performed by three experienced surgeons. The detailed surgical procedures have been illustrated in a previous report [29].

### 2.5. Statistical Analysis

Categorical parameters are shown as count with percentages (*n* (%)) and were compared using Fisher's exact test. Continuous parameters with normal distribution are shown as mean ± standard deviation (SD) and compared using Student's *t* test. In contrast, the Mann–Whitney U test was adopted for continuous parameters with non-normal distributed which were expressed as median (IQR). To determine independent risk predictors of severe PHLF in the training set, univariate analysis and multivariate logistic regression analysis were used. Only parameters with significant difference (*P* < 0.05) based on univariate regression analysis were selected for multivariate regression analysis. Calibration plots were produced to assess the performance characteristics of the nomograms. Model discrimination accuracy of nomograms, also including Child–Pugh, ALBI, MELD, APRI, PALBI, and FIB-4, for predicting PHLF grade B-C was determined using the area under the receiver operating characteristic (ROC) curve (AUC) and concordance index (C-index). A *P* value <0.05 was defined as statistically significant in all statistical analyses. All the statistical analyses were performed using Statistical Package for Social Sciences (SPSS) version 26.0 for Windows (SPSS, Chicago, IL, USA). The nomogram and calibration plots were generated using the “rms” package and “boot” method in R software (version 4.0.3).

## 3. Results

### 3.1. Patient Characteristics

The characteristics of 574 HCC cases that met the inclusion and exclusion criteria were recorded and randomly allocated to a training set (*n* = 383) and an internal validation set (*n* = 191) at a ratio of 2 : 1. A total of 574 patients were included with a mean age of 53.5 years (SD 11.7), and 507 patients (88.3%) were males. The majority of cases were infected with hepatitis B virus (83.6%), and 32.9% of patients had cirrhosis diagnosed by pathological examination, while CSPH was present in 49 out of 525 cases (8.5%). The baseline clinicopathologic characteristics of the enrolled patients are shown in Table 1. According to the preoperative PALBI score, 55 cases (14.3%) and 34 cases (17.2%) were assigned to the high PALBI group (score >−2.45) in the training and validation sets, respectively. Simultaneously, 49 (12.7%) cases and 26 (13.6%) cases were categorised as high FIB-4 group (score ≥1.45) based on the preoperative FIB-4 score (Table 1). The percentage of PHLF grade B-C was 14.8% in the total set, 15.4% in the training set, and 13.6% in the validation set (Table 1).

### 3.2. Univariate and Multivariate Analyses for PHLF Grade B-C

Univariate analysis was used to preliminarily filter independent risk indicators of PHLF grade B-C in the training set. All the variables identified as significant associations (*P* < 0.05) in univariate regression analysis were included in the multivariate regression analysis. Cirrhosis, major hepatectomy, tumour number, ascites, intraoperative blood loss (>400 mL), PALBI score (score >−2.45), and Fib-4 score (score ≥1.45) were all significantly associated (*P* < 0.05) with PHLF grade B-C (Table 2). Meanwhile, multivariate regression analysis revealed that cirrhosis (*P*=0.026, odds ratio (OR) = 2.296, 95% CI 1.102–4.786), major hepatectomy (*P*=0.031, OR = 2.211, 95% CI 1.077–4.542), ascites (*P*=0.014, OR = 3.588, 95% CI 1.299–9.913), intraoperative blood loss (>400 mL) (*P* < 0.001, OR = 4.683, 95% CI 2.281–9.616), PALBI score (score >−2.45) (*P*=0.005, OR = 3.609, 95% CI 1.486–8.764), and FIB-4 score (score ≥1.45) (*P* < 0.001, OR = 5.627, 95% CI 2.077–13.351) were the independent risk factors associated with PHLF grade B-C (Table 2).

### 3.3. Diagnostic Nomograms for PHLF Grade B-C

**Figure 1:**
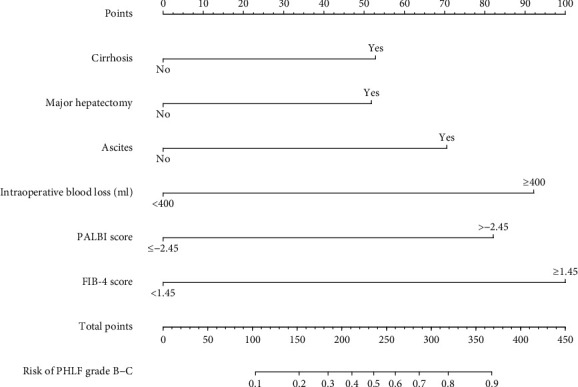
The nomogram model for predicting PHLF grade B-C in patients with HCC.

**Figure 2:**
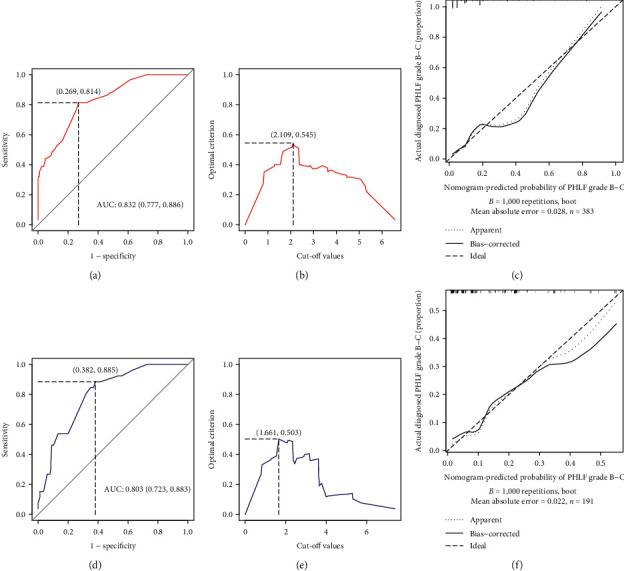
ROC curve for the constructed nomogram model for predicting PHLF grade B-C: (a) training set; (d) validation set. The optimal cut-off value (highest Youden index) for the nomogram model for predicting PHLF grade B-C: (b) training set; (e) validation set. Calibration plots show the relationship between the predicted probabilities based on the nomogram and actual values: (c) training set; (f) validation set.

**Figure 3:**
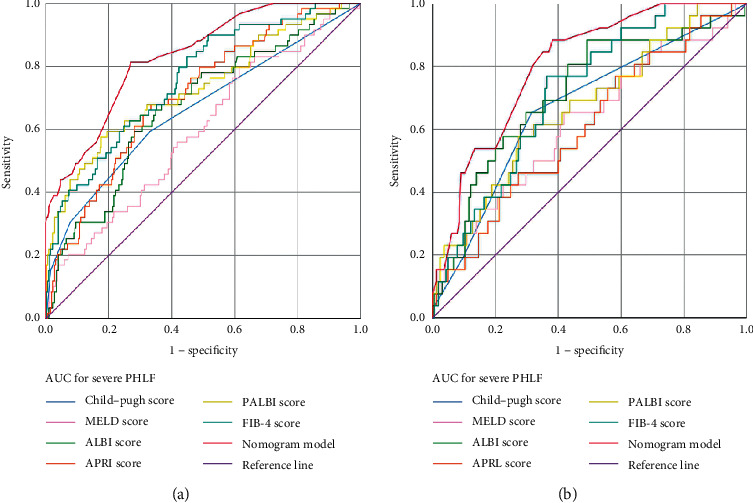
Receiver operating characteristic curves of nomogram models and Child–Pugh, the model for end-stage liver disease (MELD) score, albumin-bilirubin (ALBI), aspartate transaminase to platelet ratio index (APRI), platelet-albumin-bilirubin (PALBI) score, and fibrosis-4 score (FIB-4) for predicting PHLF grade B-C: (a) training set; (b) validation set.

Based on the results of the multivariate regression analysis (Table 2), we constructed a nomogram for predicting PHLF grade B-C using the significant independent risk factors identified in the multivariable analysis (Figure 1). ROC analysis and calibration plot were used to assess the predictive accuracy of the PHLF grade B-C nomogram. The area under the ROC curve (AUROC) of the nomogram in the training set was 0.832 (95% CI 0.777–0.886) (Figure 2(a)). Moreover, the AUROC of the nomogram had a significantly higher performance than the Child–Pugh score (0.662, 95% CI 0.580–0.745, *P* < 0.001), MELD score (0.595, 95% CI 0.516–0.674, *P*=0.020), ALBI score (0.673, 95% CI 0.597–0.748, *P* < 0.001), APRI score (0.707, 95% CI 0.638–0.777, *P* < 0.001), PALBI score (0.731, 95% CI 0.653–0.808, *P* < 0.001), or FIB-4 score (0.758, 95% CI 0.692–0.824, *P* < 0.001) (Figure 3(a) and Table 3). The optimal cut-off value (highest Youden index) of the nomogram score to predict PHLF grade B-C in the training set was determined to be 2.109 (Figure 2(b) and Table 4); the C-index, sensitivity, specificity, positive predictive values (PPV), and negative predictive values (NPV) were 0.832, 0.813, 0.731, 35.5%, and 95.5%, respectively (Table 4). The calibration curves for the probability of PHLF grade B-C showed a good correlation between prediction by nomogram and actual observation in the training set (Figure 2(c)).

### 3.4. Assessment of the Nomogram in the Validation Set

The background characteristics of the 191 patients in the validation set are also described in Table 1. The AUROC of the nomogram in the validation set for predicting PHLF grade B-C was 0.803 (95% CI 0.723–0.883) (Figure 2(c)), which had a greater discriminatory performance than other scoring models: Child–Pugh score (0.669, 95% CI 0.555–0.783, *P*=0.006), MELD score (0.621, 95% CI 0.499–0.742, *P*=0.048), ALBI score (0.715, 95% CI 0.606–0.825, *P* < 0.001), APRI score (0.604, 95% CI 0.487–0.720, *P*=0.084), PALBI score (0.669, 95% CI 0.557–0.782, *P*=0.006), and FIB-4 score (0.716, 95% CI 0.623–0.809, *P* < 0.001) (Figure 3(b) and Table 3). The optimal cut-off value (highest Youden index) of the nomogram score to predict PHLF grade B-C in the validation set was determined to be 1.661 (Figure 2(e) and Table 4). In the validation set, the C-index, sensitivity, specificity, PPV, and NPV for the diagnosis of PHLF grade B-C were 0.808, 0.884, 0.618, 26.7%, and 97.1%, respectively (Table 4). The calibration curves in the validation set were similar to those in the training set (Figure 2(f)).

## 4. Discussion

Preoperative liver functional reserve and fibrosis are critical for surgeons to establish a treatment plan and predicting PHLF grade B-C in HCC patients undergoing hepatectomy. In the present study, we developed and validated a nomogram based on two essential noninvasive liver reserve and fibrosis models (PALBI and FIB-4) in estimating PHLF grade B-C among HCC patients. By utilising AUROC analysis, a graphic and convenient tool demonstrated good capability in terms of prediction of PHLF grade B-C compared with other conventional liver function models including the Child–Pugh grade, MELD, and APRI score and showed a good correlation between prediction by nomogram and actual observation in the training and validation sets. PHLF is a dreadful complication that may cause considerable preoperative death, and its prediction warrants further research and exploration. Therefore, the keys to successful outcome include selection of surgical techniques, confirmatory surgery, selection of special perioperative care for patients with possibility of PHLF occurrence, and its severity estimation [4]. The incidence of PHLF grade B-C in our institution was 14.8%, which is consistent with what has been published previously in China in the literature [30] (14.6%, definition criteria: ISGLS). Nevertheless, foreign literature published by Prodeau et al. [4] exhibited a higher PHLF grade B-C incidence in HCC patients with cirrhosis (38%, definition criteria: ISGLS), which may illustrate that cirrhosis is the significant factor to improve the PHLF incidence. This was similar to the results of our study (23.8% in cirrhosis set). The subtle differences of the incidence may be due to the different preoperative evaluations, surgical techniques and postoperative care between home and abroad.

The function of a remaining healthy liver is strongly associated with developing PHLF [31]. For many years, Child–Pugh grade and ALBI grade have been widely utilised assessment tools for functional liver reserve before liver resection. However, Child–Pugh grade integrated ascites and hepatic encephalopathy, which will cause subjective errors and ultimate leads to a prediction performance decline in PHLF. Although the ALBI grade has exhibited optimal prediction performance in PHLF and was developed using numerous objective indices, its discrimination efficacy does not include important PHLF-related risk factors such as the PLT count [32]. Recently, a novel nomogram based on preoperative and postoperative prediction models did not consider PLT count [33]. The proposed PALBI could compensate for the shortcomings of ALBI grading. In our study, the ROC analysis revealed that the PALBI grade had better accuracy in predicting PHLF in the training set than the Child–Pugh or the ALBI grade, and the results were consistent with a previous study [34]. In contrast, Ye et al. [30] and Xu et al. [35] derived opposite conclusions: the ALBI grade showed a larger AUROC curve for predicting PHLF in HCC patients than PALBI and Child–Pugh grade, and the same results were also observed in the validation set from the present study. Therefore, much controversy remains regarding the predictive value between PALBI and ALBI in HCC patients with PHLF, which warrants further research and larger datasets. Our study is consistent with previous findings, which demonstrated that the PLT count is the significant factor for predicting PHLF. Hence, we focused on the analysis of predictive ability of high PALBI grade (score >−2.45) in predicting PHLF. As expected, high PALBI grade is a crucial variable in our predictive nomogram.

Liver fibrosis is correlated with end-stage cirrhosis and HCC [36], and fibrosis staging, as an assessment tool, may effectively estimate prognosis and the treatment efficacy of patients with chronic hepatitis virus infection [37]. Therefore, accurate presurgical assessment of HCC patients with liver fibrosis was essential for the patient's long-term prognosis. Although a liver biopsy is officially recommended as the gold standard for staging fibrosis [38], the invasiveness, cost, and inevitable sampling errors have restricted its clinical application [39, 40]. Hence, a variety of noninvasive tools such as FIB-4 and APRI have been approved by the World Health Organization for evaluating significant fibrosis [41]. The FIB-4 and APRI scores all achieved good predictive performances in predicting PHLF grade B-C in both training and validation sets. Moreover, ROC analysis showed the AUROC curves of FIB-4 were larger than that of APRI (training set: 0.758 vs 0.707; validation set: 0.716 vs 0.604). We incorporated the high FIB-4 grade (score ≥1.45) into the multivariate regression analysis, and the results demonstrated that high FIB-4 grade was an independent risk factor for predicting PHLF grade B-C. With the above approach, we developed nomogram and explored its capacity in predicting PHLF grade B-C.

Increasing research has shown that patients presenting major hepatectomy and intraoperative blood loss were factors strongly related to the risk of PHLF [4, 33]. The balance of remnant liver parenchyma volume and oncologic characterisation have been maintained in clinical practice. Major hepatectomy caused by anatomical resection can provide patients with a superior prognosis, but it seems to result in an increased risk of PHLF, especially in HCC patients with cirrhosis or hepatic fibrosis [42]. Prodeau et al. [4] collected data from 343 patients and revealed that increased blood loss was associated with a higher risk of PHLF. The formation of ascites is closely related to liver function, which is one of the reasons why it is included in the nomogram. In keeping with previous findings, major hepatectomy, intraoperative blood loss (>400 mL), and ascites were significant risk predictors for the development of PHLF grade B-C in the multivariable logistic regression analysis. Although several previous reports have proposed that ICG clearance can predict the development of PHLF after hepatic resection [43], ICG clearance did not lead to positive results that significantly predicted PHLF [44]. Therefore, several previous researches have revealed these three indicators were predictors of PHLF. Rather than adding ICG clearance to the nomogram we developed, we incorporated the major hepatectomy, intraoperative blood loss (>400 mL), and ascites reflecting liver reserve function into our nomogram model.

There were several limitations to the present study. First, as the incidence of hepatitis B virus (HBV) infections in our country has increased, the majority of the patients (83.6%) in this study had experienced chronic HBV infection. As such, the universality of the novel model needs to explore different etiological populations with cirrhosis and HCC. Second, this research was based on data from a single centre and retrospective study, which may cause selection bias. Furthermore, the sample size was relatively small, and the clinical application of our novel nomogram requires independent external and prospective multicentre studies with large dates for optimal validation. Third, postoperative future liver remnant volume was not considered, which might be taken into further research to promote the accuracy value of predicting PHLF.

## 5. Conclusion

In the current study, the results demonstrated that the high PALBI grade (score >−2.45) and high FIB-4 grade (≥1.45) were independently associated with PHLF grade B-C by multivariate regression analysis. The diagnostic nomogram model proposed herein based on four essential clinical variables (cirrhosis, major hepatectomy, ascites, and intraoperative blood loss) and two noninvasive liver reserve and fibrosis model (high PALBI grade and high FIB-4 grade) had good performance for suitably predicting PHLF grade B-C in HCC patients, in contrast to the currently available conventional noninvasive liver reserve and fibrosis (Child–Pugh, MELD, ALBI, and APRI) models in both training and validation sets. This model may contribute to facilitate the preoperative workup of clinicians in predicting the probability of risk of PHLF in HCC patients after hepatectomy.

## Figures and Tables

**Table 1:** Base characteristics of patients in the training set (*n* = 383) and validation set (*n* = 191).

Variable	Total	Training set (*n* = 383)	Validation set (*n* = 191)	*P* value
Sex (male vs. female)	507/67 (88.3%/11.6%)	338/45 (88.2%/11.7%)	169/22 (88.4%/11.5%)	0.384
Age (years)	53.57 ± 11.70	53.92 ± 11.51	53.01 ± 12.01	0.375
Platelet count (×10^9^/L)	185.5 (137.7, 246.0)	182 (138.0, 244.0)	192.0 (137.0, 247.8)	0.699
Serum albumin (g/L)	37.5 ± 4.6	37.5 ± 4.7	37.5 ± 4.4	0.984
INR	1.07 (1.01, 1.13)	1.07 (1.02, 1.13)	1.05 (0.99, 1.10)	0.001
Serum ALT (U/L)	33.0 (22.0, 47.0)	32.0 (22.0, 48.0)	34.0 (22.7, 43.0)	0.896
Serum AST (U/L)	37.0 (26.9, 54.0)	37.0 (26.0, 56.0)	35.0 (28.0, 51.0)	0.625
Serum bilirubin (*μ*mol/L)	15.2 (11.7, 19.5)	14.5 (11.3, 19.2)	16.5 (12.6, 20.00)	0.002
Serum ALP (IU/L)	89.4 (68.0, 115.0)	90.0 (68.0, 115.0)	89.0 (67.0, 115.0)	0.850
Serum *γ*-GGT (IU/L)	57.0 (32.0, 115.3)	61.0 (33.0, 117.8)	53.6 (31.00, 113.0)	0.267
Serum CR (mmol/L)	78.0 (68.0, 89.0)	77.4 (67.6, 89.0)	77.4 (67.6, 89.0)	0.719
AFP (≥400/<400 (ng/mL))	177/397 (30.8%/69.1)	110/273 (28.7/71.2)	67/12 (35%/65.9%)	0.126
Cirrhosis (yes/no)	189/385 (32.9%/69.0%)	128/255 (33.4%/66.5%)	61/130 (31.9%/68.0)	0.778
CSPH (yes/no)	49/525 (8.5%/91.4%)	31/352 (8%/91.9%)	18/173 (9.4%/90.5%)	0.635
Positive HBsAg (yes/no)	480/90 (83.6%/16.3%)	317/66 (82.7%/17.2%)	163/28 (85.3%/14.6%)	0.240
Tumour number (multiple/single)	101/473 (17.5%/82.4%)	75/308 (19.5%/80.4%)	26/165 (13.6%/86.3%)	0.082
Ascites (yes/no)	45/529 (7.8%/92.1%)	28/355 (7.3%92.6%)	17/174 (8.9%/91.0%)	0.513
Tumour size (cm)	5.0 (2.8, 8.0)	4.5 (2.5, 8.0)	5.5 (3.1, 8.5)	0.078
Blood loss (mL)	265 (100, 700)	250 (100, 600)	300 (100, 800)	0.219
Major hepatectomy (yes/no)	210/364 (36.5%/63.4%)	139/244 (36.2%/63.7%)	71/120 (37.1%/62.8%)	0.854
Microvascular invasion (yes/no)	190/384 (33.1%/66.8%)	113/270 (29.5%/70.4%)	79/112 (41.3%/58.6%)	0.005
PHLF (0/A vs. B/C)	489/85 (85.1%/14.8%)	324/59 (84.5%/15.4%)	165/26 (86.3%/13.6%)	0.619
Child–Pugh grade (A/B)	510/64 (88.8%/11.1%)	340/43 (88.7%/11.2%)	139/21 (89.0%/10.9%)	0.560
ALBI score	−2.41 ± 0.40	−2.42 ± 0.42	−2.39 ± 0.38	0.400
MELD score	5.58 (3.81, 7.42)	5.61 (3.80, 7.38)	5.52 (3.84, 7.47)	0.582
APRI score	0.53 (0.32, 0.86)	0.54 (0.32, 0.87)	0.53 (0.32, 0.86)	0.903
PALBI score (≤−2.53/>2.53)	−2.98 (−3.21–2.68) 485/89 (84.4%/15.5%)	−2.99 (−3.23–2.69) 328/55 (85.6%/14.3%)	−2.94 (-3.17–2.64) 157/34 (82.1%/17.8%)	0.151
FIB-4 score (<1.45/≥1.45)	0.65 (0.42 1.04) 499/75 (86.9%/13.0%)	0.67 (0.43 1.05) 334/49 (87.2%/12.7%)	0.61 (0.39 0.99) 165/26 (86.3%/13.6%)	0.410

Abbreviations: INR, international normalised ratio; ALT, alanine aminotransferase; AST, aspartate aminotransferase; ALP, alkaline phosphatase; *γ*-GGT, *γ*-glutamyl transpeptidase; CR, creatinine; AFP, alpha fetoprotein; CSPH, clinically significant portal hypertension; HbsAg, hepatitis B surface antigen; PHLF, postoperative liver failure; ALBI, albumin-bilirubin; MELD, model for end-stage liver disease; APRI, aspartate aminotransferase to platelet ratio index; PALBI, platelet-albumin-bilirubin; FIB-4, fibrosis-4 index.

**Table 2:** Univariate and multivariable analyses to identify factors predicting PHLF grade B-C.

Variables	Univariate logistic regression			Multivariable logistic regression		
	*β*	Odds ratio	*P* value	*β*	Odds ratio	*P* value
Age (years)	0.024	1.025	0.056			
Sex (male)	0.365	1.440	0.365			
Serum ALP (IU/L)	<0.001	1.000	0.894			
Serum *γ*-GGT (IU/L)	0.001	1.001	0.069			
Positive HBsAg	−0.224	0.783	0.493			
Cirrhosis	1.118	3.060	<**0.001**	0.831	2.296	**0.026**
Major hepatectomy	0.795	2.214	**0.005**	0.794	2.211	**0.031**
AFP (>400 ng/mL)	0.005	1.005	0.986			
Tumour size (>5 cm)	0.512	1.688	0.073			
Tumour number (multiple)	0.920	2.508	**0.003**	0.649	1.913	0.090
Ascites	1.420	4.138	**0.001**	1.278	3.588	**0.014**
Intraoperative blood loss (>400 mL)	1.355	3.878	<**0.001**	1.544	4.683	<**0.001**
PALBI score (>−2.53)	2.081	8.015	<**0.001**	1.283	3.609	**0.005**
FIB-4 score (≥1.45)	2.104	8.201	<**0.001**	1.661	2.077	<**0.001**

Abbreviations: ALP, alkaline phosphatase; *γ*-GGT, *γ*-glutamyl transpeptidase; HbsAg, hepatitis B surface antigen; AFP, alpha fetoprotein; PALBI, platelet-albumin-bilirubin; FIB-4, fibrosis-4 index.

**Table 3:** Discriminatory performance of conventional scores and the nomogram for predicting PHLF grade B-C.

	Training set (*n* = 383)			Validation set (*n* = 191)		
	AUC	95% CI	*P* value	AUC	95% CI	*P* value
Child–Pugh	0.662	0.580–0.745	<0.001	0.669	0.555–0.783	0.006
MELD	0.595	0.516–0.674	=0.020	0.621	0.499–0.742	0.048
ALBI	0.673	0.597–0.748	<0.001	0.715	0.606–0.825	<0.001
APRI	0.707	0.638–0.777	<0.001	0.604	0.487–0.720	0.089
PALBI	0.731	0.653–0.808	<0.001	0.669	0.558–0.782	0.006
FIB-4	0.758	0.692–0.824	<0.001	0.716	0.623–0.809	<0.001
Nomogram	0.832	0.777–0.886	<0.001	0.803	0.723–0.883	<0.001

Abbreviations: MELD, model for end-stage liver disease; ALBI, albumin-bilirubin; APRI, aspartate aminotransferase to platelet ratio index; PALBI, platelet-albumin-bilirubin; FIB-4, fibrosis-4 index.

**Table 4:** Accuracy of the prediction score of the nomogram for evaluating the risk of PHLF grade B/C incidence.

Variable	Training set (*n* = 383)		Validation set (*n* = 191)	
	Value	95% CI	Value	95% CI
Area under ROC curve	0.832	0.777–0886	0.803	0.723–0.883
Cut-off score	2.109	—	1.66	—
C-index	0.832	—	0.808	—
Sensitivity (%)	0.813	0.690–0.903	0.884	0.698–0.975
Specificity (%)	0.731	0.679–0.778	0.618	0.539–0.692
Positive predictive value (%)	35.5	30.0–54.0	26.7	20.8–65.5
Negative predictive value (%)	95.5	91.6–96.5	97.1	91.1–97.9
Positive likelihood ratio	3.02	2.43–3.76	2.31	1.82–2.94
Negative likelihood ratio	0.25	0.14–0.43	0.18	0.06–0.54

## Data Availability

The datasets generated and/or analysed during the current study are available from the corresponding author upon reasonable request.

## References

[1] Uttley L., Indave B. I., Hyde C., White V., Lokuhetty D., Cree I. (2020). Invited commentary- WHO classification of tumours: how should tumors be classified? expert consensus, systematic reviews or both?. *International Journal of Cancer*.

[2] Vibert E., Schwartz M., Olthoff K. M. (2020). Advances in resection and transplantation for hepatocellular carcinoma. *Journal of Hepatology*.

[3] Kim Y. S., Shin S. W. (2019). Hepatocellular carcinoma. *New England Journal of Medicine*.

[4] Prodeau M., Drumez E., Duhamel A. (2019). An ordinal model to predict the risk of symptomatic liver failure in patients with cirrhosis undergoing hepatectomy. *Journal of Hepatology*.

[5] Melloul E., Hübner M., Scott M. (2016). Guidelines for perioperative care for liver surgery: enhanced recovery after surgery (ERAS) society recommendations. *World Journal of Surgery*.

[6] Citterio D., Facciorusso A., Sposito C., Rota R., Bhoori S., Mazzaferro V. (2016). Hierarchic interaction of factors associated with liver decompensation after resection for hepatocellular carcinoma. *JAMA Surgery*.

[7] Douard R., Lentschener C., Ozier Y., Dousset B. (2009). Operative risks of digestive surgery in cirrhotic patients. *Gastroentérologie Clinique et Biologique*.

[8] Zou H., Yang X., Li Q.-L., Zhou Q.-X., Xiong L., Wen Y. (2018). A comparative study of albumin-bilirubin score with Child-Pugh score, model for end-stage liver disease score and indocyanine green R15 in predicting posthepatectomy liver failure for hepatocellular carcinoma patients. *Digestive Diseases*.

[9] Ross S. W., Seshadri R., Walters A. L. (2016). Mortality in hepatectomy: model for end-stage liver disease as a predictor of death using the national surgical quality improvement program database. *Surgery*.

[10] Bruix J., Sherman M., American Association for the Study of Liver Diseases (2011). Management of hepatocellular carcinoma: an update. *Hepatology*.

[11] Ichikawa T., Uenishi T., Takemura S. (2009). A simple, noninvasively determined index predicting hepatic failure following liver resection for hepatocellular carcinoma. *Journal of Hepato-Biliary-Pancreatic Surgery*.

[12] Xiao G., Yang J., Yan L. (2015). Comparison of diagnostic accuracy of aspartate aminotransferase to platelet ratio index and fibrosis-4 index for detecting liver fibrosis in adult patients with chronic hepatitis B virus infection: a systemic review and meta-analysis. *Hepatology*.

[13] Johnson P. J., Berhane S., Kagebayashi C. (2015). Assessment of liver function in patients with hepatocellular carcinoma: a new evidence-based approach-the ALBI grade. *Journal of Clinical Oncology*.

[14] Wiesner R., Edwards E., Freeman R. (2003). Model for end-stage liver disease (MELD) and allocation of donor livers. *Gastroenterology*.

[15] Malinchoc M., Kamath P. S., Gordon F. D., Peine C. J., Rank J., Ter Borg P. C. J. (2000). A model to predict poor survival in patients undergoing transjugular intrahepatic portosystemic shunts. *Hepatology*.

[16] Naqvi I. H., Talib A., Mahmood K., Abidi R., Zehra Rizvi S. N. (2019). The ability of the new ALBI scoring in predicting mortality, complications and prognostic comparison among cirrhotics. *Gastroenterology Review*.

[17] Nagorney D. M., Kamath P. S. (2006). Predictive indices of morbidity and mortality after liver resection. *Annals of Surgery*.

[18] Schreckenbach T., Liese J., Bechstein W. O., Moench C. (2012). Posthepatectomy liver failure. *Digestive Surgery*.

[19] Zheng Y.-W., Wang K.-P., Zhou J.-J. (2018). Portal hypertension predicts short-term and long-term outcomes after hepatectomy in hepatocellular carcinoma patients. *Scandinavian Journal of Gastroenterology*.

[20] Lee S. K., Song M. J., Kim S. H., Park M. (2019). Comparing various scoring system for predicting overall survival according to treatment modalities in hepatocellular carcinoma focused on platelet-albumin-bilirubin (PALBI) and albumin-bilirubin (ALBI) grade: a nationwide cohort study. *PLoS ONE*.

[21] Hansmann J., Evers M. J., Bui J. T. (2017). Albumin-bilirubin and platelet-albumin-bilirubin grades accurately predict overall survival in high-risk patients undergoing conventional transarterial chemoembolization for hepatocellular carcinoma. *Journal of Vascular and Interventional Radiology*.

[22] Llovet J. M., Zucman-Rossi J., Pikarsky E. (2016). Hepatocellular carcinoma. *Nature Reviews Disease Primers*.

[23] Rahbari N. N., Garden O. J., Padbury R. (2011). Posthepatectomy liver failure: a definition and grading by the international study group of liver surgery (ISGLS). *Surgery*.

[24] Berzigotti A., Reig M., Abraldes J. G., Bosch J., Bruix J. (2015). Portal hypertension and the outcome of surgery for hepatocellular carcinoma in compensated cirrhosis: a systematic review and meta-analysis. *Hepatology*.

[25] Pol B., Campan P., Hardwigsen J., Botti G., Pons J., Le Treut Y. P. (1999). Morbidity of major hepatic resections: a 100-case prospective study. *The European Journal of Surgery = Acta Chirurgica*.

[26] Moore K. P., Aithal G. P. (2006). Guidelines on the management of ascites in cirrhosis. *Gut Microbiota*.

[27] Pugh R. N. H., Murray-Lyon I. M., Dawson J. L., Pietroni M. C., Williams R. (1973). Transection of the oesophagus for bleeding oesophageal varices. *British Journal of Surgery*.

[28] Vallet-Pichard A., Mallet V., Nalpas B. (2007). FIB-4: an inexpensive and accurate marker of fibrosis in HCV infection. comparison with liver biopsy and fibrotest. *Hepatology*.

[29] Zhong J.-H., Ke Y., Gong W.-F. (2014). Hepatic resection associated with good survival for selected patients with intermediate and advanced-stage hepatocellular carcinoma. *Annals of Surgery*.

[30] Ye J. Z., Mai R. Y., Guo W. X. (2020). Nomogram for prediction of the international study group of liver surgery (ISGLS) grade B/C posthepatectomy liver failure in HBV-related hepatocellular carcinoma patients: an external validation and prospective application study. *BMC Cancer*.

[31] Shen Y.-N., Zheng M.-L., Guo C.-X. (2018). The role of imaging in prediction of post-hepatectomy liver failure. *Clinical Imaging*.

[32] Tomimaru Y., Eguchi H., Gotoh K. (2016). Platelet count is more useful for predicting posthepatectomy liver failure at surgery for hepatocellular carcinoma than indocyanine green clearance test. *Journal of Surgical Oncology*.

[33] Shi J. Y., Sun L. Y., Quan B. (2020). A novel online calculator based on noninvasive markers (ALBI and APRI) for predicting post-hepatectomy liver failure in patients with hepatocellular carcinoma. *Clinics and Research in Hepatology and Gastroenterology*.

[34] Lu L.-H., Zhang Y.-F., Mu-Yan C. (2019). Platelet-albumin-bilirubin grade: risk stratification of liver failure, prognosis after resection for hepatocellular carcinoma. *Digestive and Liver Disease*.

[35] Xu Y., Hu X., Li J., Dong R., Bai X. (2020). An improved scoring system based on PALBI in predicting post-hepatectomy liver failure outcomes. *Digital Distribution*.

[36] Sun M., Kisseleva T. (2015). Reversibility of liver fibrosis. *Clinics and Research in Hepatology and Gastroenterology*.

[37] European Association for the Study of the Liver (2017). Electronic address eee, European association for the study of the L. EASL 2017 clinical practice guidelines on the management of hepatitis B virus infection. *Journal of Hepatology*.

[38] Rockey D. C., Caldwell S. H., Goodman Z. D., Nelson R. C., Smith A. D. (2009). Liver biopsy. *Hepatology*.

[39] Strassburg C., Manns M. (2006). Approaches to liver biopsy techniques-revisited. *Seminars in Liver Disease*.

[40] Regev A., Berho M., Jeffers L. J. (2002). Sampling error and intraobserver variation in liver biopsy in patients with chronic HCV infection. *The American Journal of Gastroenterology*.

[41] Tang Y., Zhang Z., Song X. (2020). Tumor-derived exosomal miR-620 as a diagnostic biomarker in non-small-cell lung cancer. *Journal of Oncology*.

[42] Dong S., Wang Z., Wu L., Qu Z. (2016). Effect of surgical margin in R0 hepatectomy on recurrence-free survival of patients with solitary hepatocellular carcinomas without macroscopic vascular invasion. *Medicine (Baltimore)*.

[43] Kim S. U., Ahn S. H., Park J. Y. (2008). Prediction of postoperative hepatic insufficiency by liver stiffness measurement (FibroScan(R)) before curative resection of hepatocellular carcinoma: a pilot study. *Hepatology International*.

[44] Wong J. S.-W., Wong G. L.-H., Chan A. W.-H. (2013). Liver stiffness measurement by transient elastography as a predictor on posthepatectomy outcomes. *Annals of Surgery*.

